# Effects of Hand Positions During Video Head-Impulse Test (vHIT) in Patients With Unilateral Vestibular Neuritis

**DOI:** 10.3389/fneur.2018.00531

**Published:** 2018-08-13

**Authors:** Wei Fu, Feng He, Ruoqi Zhao, Dong Wei, Ya Bai, XiaoMing Wang, JunLiang Han

**Affiliations:** ^1^Department of Geriatrics, Xijing Hospital, Fourth Military Medical University, Xi'an, China; ^2^Department of Neurology, Xijing Hospital, Fourth Military Medical University, Xi'an, China; ^3^Queen Mary College, Nanchang University, Nanchang, China

**Keywords:** video head-impulse test, horizontal vestibulo-ocular reflex, hand position, vestibular tests, horizontal semicircular canal

## Abstract

**Background:** The aim of this study is to identify the effects of hand positions (head and jaw) on the video head-impulse test (vHIT).

**Methods:** Eighty-six healthy volunteers and sixty-seven patients with unilateral vestibular neuritis (UVN) were recruited for this study. Different hand positions (head and jaw) were used in the vHIT of horizontal semicircular canals in healthy volunteers and UVN patients. All the obtained horizontal vHIT gains were analyzed.

**Results:** It was observed that when horizontal vHIT was performed with the head hand position, the number of head impulses that produced overhigh vestibulo-ocular reflex (VOR) gains was more than that with the jaw hand position (*p* < 0.01), irrespective of whether the test was performed in healthy volunteers or UVN patients. The gains obtained were lower when the jaw hand position was used than that obtained when the head hand position was used (*p* < 0.05). However, no significant difference existed in the mean head velocity between the two hand positions (*p* > 0.05). Using the head hand position has greater a chance to elicit in UVN patients normal horizontal vHIT gains with refixation saccades than using the jaw hand position (*p* = 0.04).

**Conclusion:** The jaw hand position can increase the accuracy of vHIT in determining the lesion side.

## Introduction

The vestibule-ocular reflex (VOR) helps to stabilize the retinal image by rotating the eyes to compensate for movements of the head. The VOR has three main components: a peripheral sensory apparatus (the semicircular canals and the otolith organs), a central processing mechanism, and a motor output. It has two different physical properties. The angular VOR mediated by the semicircular canals compensates for rotation. It is primarily responsible for gaze stabilization. The linear VOR mediated by the otolith organs (saccule and utricle) compensates for translation. It is very important in situations where nearby targets are being viewed (1).

The bedside head-impulse test (bHIT) is a well-recognized clinical tool to test the VOR. It was first described by Halmagyi and Curthoys in 1988 as a test of the VOR and has since become an established bedside assessment method in the evaluation of vertigo (2). In the bHIT, a subject maintains fixation on an object straight ahead as sudden head impulses are applied in the horizontal angular plane and eye movements are observed for catch-up saccades (2). If the subject's VOR is normal, the eyes can remain focused on the fixation target during head rotations. If the VOR is insufficient, the eyes will not be able to fixate on the target during head movements. Instead, compensatory quick eye movements toward the target, called saccades, will occur (2). If these quick eye movements occur after head rotations stop, they are called overt saccades. If the quick compensatory eye movements occur during head movements, they are called covert saccades (3). When performing the bHIT, the clinician can detect overt saccades but not covert saccades. Recently, the video head-impulse test (vHIT) has been introduced to overcome this problem and to measure the VOR gain quantitatively (4). Conducting the vHIT allows the clinician to visualize the VOR in its physiological range as in search-coil measurements, which are the gold standard of the vHIT (5). Thus, the vHIT provides a new method to record eye movements and head velocity so that VOR gain reduction and refixation saccades can be analyzed quantitatively (3).

In the vHIT, the examiner who performs the head impulse test should master sufficient skills. When a horizontal vHIT is performed, the positions of the clinician's hands placed on the top of the head or on the jaw of the patient may vary among clinicians (6, 7). A few studies have been conducted that explore whether the hand positions of the examiner affect the horizontal vHIT in healthy individuals and vestibular disorder patients. Hence, in this study, we quantitatively measured vHIT gains with different hand positions (head and jaw) in healthy volunteers and unilateral vestibular neuritis (UVN) patients. In addition, we tried to identify the effects of hand positions on the vHIT.

## Methods

### Participants

86 healthy volunteers were recruited for this study. Their mean age was 42.50 ± 13.99 (ranging from 19 to 73 years). All subjects had normal hearing and vestibular function. They were devoid of any previous or current history of audiological or vestibular disorders. They did not show any abnormalities on complete neuro-otologic examination.

We identified 67 patients with UVN (mean age 46.71 ± 14.69; ranging from 28 to 77 years). All patients were examined in 2016 and 2018 at a vertigo clinic in the Department of Neurology in Xijing Hospital, Fourth Military Medical University. They were examined for vertigo, dizziness, or imbalance due to acute UVN for about 3–36 months (mean 8.4 ± 6.3 months). All patients met the clinical diagnostic criteria for UVN including sudden onset of prolonged vertigo (more than 1 day) with unidirectional spontaneous horizontal–torsional nystagmus, reduced or absent unilateral caloric response, absence of other auditory or neurologic findings, and no signs of central nervous system diseases (8).

All subjects provided written informed consents to participate in this study. The study was approved by the Institutional Review Board of Xijing Hospital, Fourth Military Medical University, where the patients were enrolled.

### vHIT with two different hand positions (head and jaw)

**Figure 1:**
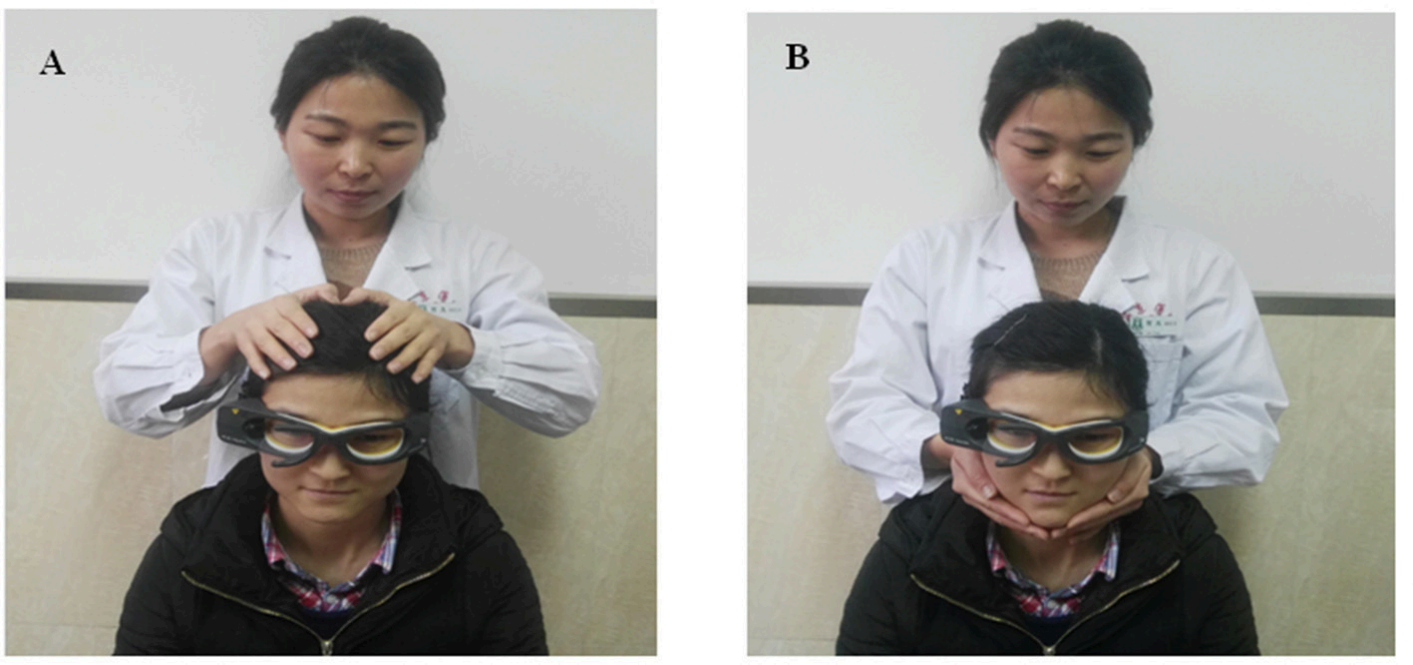
Horizontal vHITs were performed with two different hand positions (written informed consent was obtained from examiner and volunteer for publishing the image). **(A)** Head hand position: examiner's hands placed on the top of the control's head during horizontal vHIT, well away from the goggles strap. **(B)** Jaw hand position: examiner clenched participant's teeth during the thrust to reduce jaw movements and facilitate a more direct force transfer to the head.

**Figure 2:**
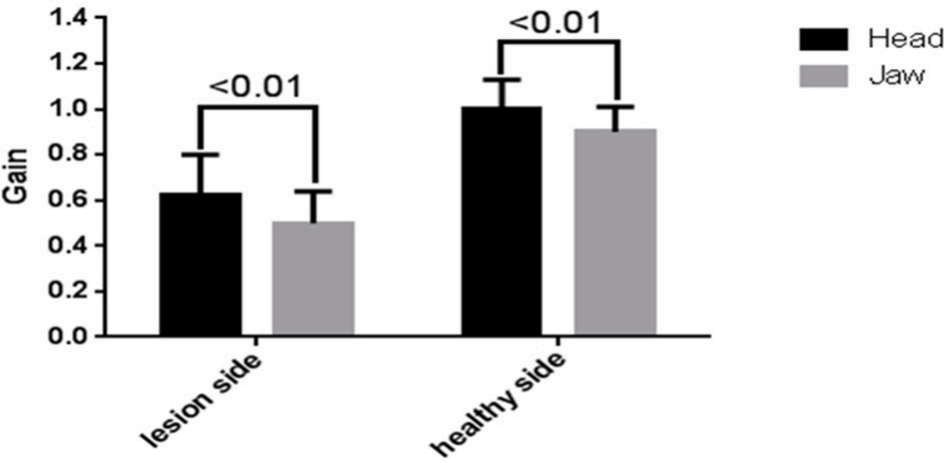
Effects of different hand positions on average measured horizontal vHIT gains for unilateral vestibular neuritis (UVN) patients. The mean horizontal vHIT gains were lower in the jaw hand position (gray) compared with the head hand position (black) on the lesion side and the healthy side.

The VOR evaluation for horizontal semicircular canals was performed with an ICS Impulse system (Otometrics, Denmark). The system includes a pair of light-weighted tight-fitting goggles on which a small video camera and a half-silvered mirror, which reflects the image of the control's right eye into the camera, are mounted. The right eye is illuminated by a low-power infrared light-emitting diode. A small sensor on the goggles measures the head movements and the camera records the eye movements. The entire system weighs about 60 g and is secured tightly to the head to minimize slippage of the goggles. When using the system to perform the test, the clinician should help the control to wear the goggles in the correct position. First, calibration is performed and the procedure of vestibulo-ocular testing is initiated. The clinician asks the control to keep staring at an earth-paralleled target 1.2 m in front. In each trial, the clinician turns the control's head to the left and right briefly and unpredictably in the horizontal plane by a small angle (approximately 10–20°) and an appropriate velocity (150–200°/s). In a full test, at least 20 impulses are randomly delivered in each direction. In our study, the head thrusts for horizontal semicircular canals were performed with two different hand positions for each participant: (1) head hand position: horizontal vHIT was performed with both hands on the top of the head, well away from the goggles strap (Figure 1A), (2) jaw hand position: the examiner clenched the participant's teeth during the thrust to reduce jaw movements and facilitated a more direct force to be transferred to the head so as to reduce movement artifacts (Figure 1B). The mean horizontal vHIT gains (ratio of eye velocity to head velocity) and the appearances of refixation saccades during and after head impulses to the right and left were the evaluated parameters. Pathological saccades were randomly registered throughout the procedure, during or after the head impulse, with peak velocities above 100°/s (9). All examinations were performed by a trained physician who is particularly skilled at neuro-otological testing and the vHIT.

### Statistical analysis

To analyze the mean horizontal vHIT gains and the significance of differences of the mean horizontal vHIT gains between two different hand positions was determined with the Student's *t*-test. The two hand positions were compared for the number of head impulses that produced overhigh VOR gains in the Chi-square tests. The differences between the numbers of the UVN patients who had normal horizontal vHIT gains but with refixation saccades in two different hand positions were evaluated by using Chi-square tests. The *p*-value for statistically significant differences was set at 0.05. Statistical analysis was performed using IBM SPSS Statistics version 19 (SPSS, Inc., Chicago, IL, USA).

## Results

### Healthy volunteers

We included 86 healthy volunteers. A total of 3,440 head impulses (1,720 rightwards and 1,720 leftwards) were measured. When horizontal vHIT was performed with both hands on the top of the head, there were overhigh VOR gains (eye velocity/head velocity >1) in 1,398 head impulses (746 rightwards and 652 leftwards) and the mean gains were 1.06 ± 0.07 (range 0.90–1.19) for the rightward turns and 1.02 ± 0.07 (range 0.88–1.23) for the leftward turns. Furthermore, the mean head velocity was 168.43 ± 14.21°/s (range 150–180°/s) in the right side and 170.75 ± 13.11°/s (range 155–185°/s) in the left side, respectively. When combining the jaw hand position, there were overhigh VOR gains (eye velocity/head velocity >1) in 879 head impulses (431 rightwards and 448 leftwards) and the mean horizontal vHIT gains for healthy participants were 1.01 ± 0.06 (range 0.87–1.13) for the right and 0.95 ± 0.06 (range 0.83–1.09) for the left. Furthermore, the mean head velocity was 169.35 ± 12.82°/s (range 150–180°/s) in the right side and 170.15 ± 14.46°/s (range 150–190°/s) in the left side, respectively. There was a significant difference in the number of head impulses that produced overhigh VOR gains between the two hand positions (*p* < 0.01). In addition, irrespective of the left or right positions, gains were lower in the jaw hand position compared with the head hand position (*p* < 0.01, Table 1). However, there was no significant difference in the mean head velocity between the two hand positions (*p* > 0.05, Table 1).

**Table 1:** Mean gains and head velocity of horizontal semicircular canals during vHIT in normal subjects (*n* = 86).

	**Head hand position**	**Jaw hand position**	***p-*value**
Left mean gain (range)	1.02 ± 0.07 (0.88–1.23)	0.95 ± 0.06 (0.83–1.09)	<0.01^*^
Right mean gain (range)	1.06 ± 0.07 (0.90–1.19)	1.01 ± 0.06 (0.87–1.13)	<0.01^*^
Left mean head velocity (range)	170.75 ± 13.11 (155–185)	170.15 ± 14.46 (150–190)	0.87^*^
Right mean head velocity (range)	168.43 ± 14.21 (150–180)	169.35 ± 12.82 (150–180)	0.75^*^

*Data are expressed as mean ± standard deviation (minimum–maximum range)*.

* *t-test*.

### Unilateral vestibular neuritis (UVN)

**Figure 3:**
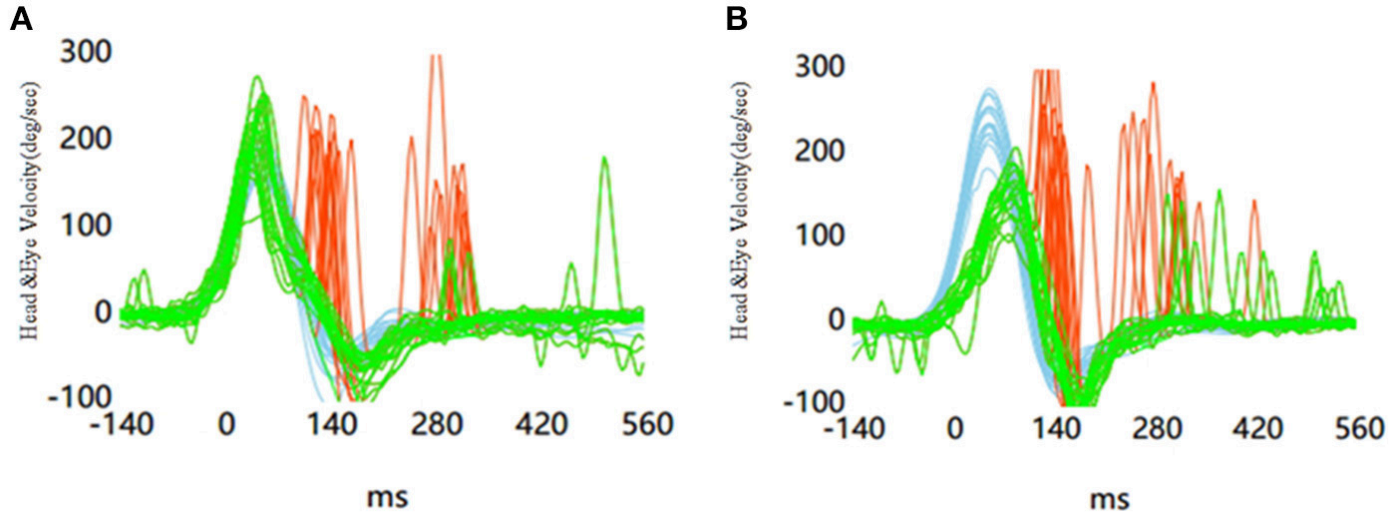
Results in the leftward vHIT in a patient with vestibular neuritis in the left side. **(A)** Horizontal vHITs were performed with head hand position. **(B)** Horizontal vHITs were performed with jaw hand position.

67 UVN patients were included in this study. A total of 2,680 head impulses (1,340 rightwards and 1,340 leftwards) were measured. There were overhigh VOR gains (eye velocity/head velocity>1) in 794 head impulses (569 healthy side and 225 lesion side) with the head hand position and 440 head impulses (311 healthy side and 129 lesion side) with the jaw hand position. There was a significant difference in the number of head impulses that produced overhigh VOR gains between the two hand positions (*p* < 0.01). The mean horizontal vHIT gains on the lesion side were 0.63 ± 0.20 (range 0.27–1.15) with the head hand position and 0.52 ± 0.18 (range 0.12–1.04) with the jaw hand position. The mean horizontal vHIT gains on the healthy side were 0.99 ± 0.10 (range 0.81–1.18) with the head hand position and 0.92 ± 0.09 (range 0.80–1.15) with the jaw hand position. The horizontal vHIT gains were lower with the jaw hand position when compared with the head hand position, irrespective of whether they were on the lesion side or on the healthy side (*p* < 0.01, Table 2, Figure 2). Furthermore, the mean head velocity on the lesion side was 166.22 ± 11.32°/s (range 150–180°/s) with the head hand position and 168.25 ± 12.50°/s (range 155–185°/s) with the jaw hand position, respectively. The mean head velocity on the healthy side was 165.49 ± 13.27°/s (range 150–180°/s) with the head hand position and 167.50 ± 15.36°/s (range 155–190°/s) with the jaw hand position, respectively. There was no significant difference in the mean head velocity between the two hand positions (*p* > 0.05, Table 2). We set an abnormal criteria for gain values below 0.8 in accordance with some previous reports (5, 9). When horizontal vHIT was performed with both hands on the top of the head in all UVN patients, 21 patients displayed refixation saccades with normal gain values. When combining the jaw hand position in all UVN patients, 11 patients displayed refixation saccades with normal gain values and all the 11 patients were included in 21 patients. Using the head hand position has a greater chance to elicit in UVN patients normal horizontal vHIT gains but with refixation saccades than the using jaw hand position (*p* = 0.04, Figure 3).

**Table 2:** Mean gains and head velocity of horizontal semicircular canals during vHIT in unilateral vestibular neuritis patients (*n* = 67).

	**Head hand position**	**Jaw hand position**	***p*-value**
Lesion-side mean gain (range)	0.63 ± 0.20 (0.27–1.15)	0.52 ± 0.18 (0.12–1.04)	<0.01^*^
Healthy-side mean gain (range)	0.99 ± 0.10 (0.81–1.18)	0.92 ± 0.09 (0.80–1.15)	<0.01^*^
Lesion-side mean head velocity (range)	166.22 ± 11.32 (150–180)	168.25 ± 12.50 (155–185)	0.42^*^
Healthy-side mean head velocity (range)	165.49 ± 13.27 (150–180)	167.50 ± 15.36 (155–190)	0.38^*^

*Data are expressed as mean ± standard deviation (minimum–maximum range)*.

* *t-test*.

## Discussion

The introduction of the vHIT provides a new dimension to vestibular diagnostics. The registration and measurement of the eye responses to rapid head thrusts during the vHIT is an innovation in the evaluation of dizziness in patients and can be routinely used in clinical settings (10). The vHIT is more sensitive in detecting unilateral vestibulopathy than the bHIT (11). Moreover, the vHIT is a quantitative method for measuring the severity of unilateral vestibular weakness, in that a VOR gain for each ear can be an important indicator of vestibular impairment. In the vHIT technique, the head-impulse test requires the examiner who performs the head movements to have sufficient skills. This is an important precondition in the question of whether an exact gain value can be affected by the hand positions (head and jaw). Therefore, the aim of this study was to identify the effects of hand positions (head and jaw) on the vHIT in healthy volunteers and UVN patients.

In our study, we found that the mean horizontal vHIT gains were obviously affected by the hand positions in healthy volunteers and UVN patients. There was a significant decrease in the VOR gains when using the jaw hand position compared with the head hand position. Leh et al. reported that hand positions did not lead to significant differences in horizontal vHIT gains. It may not be sufficient to detect a significant difference as the analysis was based only on 9 subjects (12). After testing 40 healthy adults, Patterson et al. found that placing hands on the top of the head resulted in significantly higher horizontal vHIT gains than placing them on the chin (13). This is consistent with the result obtained by us in the case of a healthy individual. However, it was unknown whether the hand positions affected the vHIT in vestibular diseases. Therefore, our study included 67 UVN patients in order to make a comparison between the effects of the head hand position and the jaw hand position on vHIT results. Similarly, the mean horizontal vHIT gains were higher with the head hand position than with the jaw hand position in UVN patients. In addition, in this study, when horizontal vHIT was performed with the head hand position, it sometimes produced more high VOR gains (eye velocity/head velocity >1) when compared with the jaw hand position in healthy volunteers and UVN patients. Some reasons can explain these outcomes. First, due to the high-speed acceleration of the head thrusts (2,500–3,000 degrees/s^2^) (14), the vHIT goggles may slip from the face or skull (15), which, in turn, might induce overhigh VOR gains. Furthermore, the slippage of the goggles is especially problematic in Asian subjects, as the majority of the vHIT devices are designed based on the facial features of Caucasian individuals. In general, Asians have a lower nasal dorsum. The goggles tend to float over the nose, but tightening the strap can result in excessive pressure on the lateral eye rims. Suh et al. quantitatively measured the tightness of the strap using different pressures (25, 35, and 45 cm H_2_O) and tried to identify slippage-induced artifacts for each pressure. At 60 and 80 ms, the gain was high for the loose (25 cm H_2_O) and the tight (35 cm H_2_O) conditions compared with the very tight condition (45 cm H_2_O) (16). They recommended that monitoring the pressure of the strap tightness might be a solution for minimizing this slippage. However, the hand position was not described in this study. In our study, we evaluated the differences between hand positions (head and jaw) during horizontal vHIT in healthy Asians individuals and UVN patients. Our findings revealed a significant higher mean horizontal vHIT gain when using the head hand position compared with the jaw hand position. In addition, we found that the head hand position would make the hair move, so that the goggles might slip slightly from the face or skull. Using the jaw hand position could avoid hair contact and reduce the slippage of the goggles. Moreover, age-related skin differences might introduce more goggle slippage in the head hand position than the jaw hand position (13). The reason is that the skin of the scalp becomes thinner, stiffer, less tense, and less flexible with the increase of age (17). In our study, two groups of participants were recruited; healthy volunteers were between the ages of 19 and 73 years of age and UVN patients were between 28 and 77 years of age. The vHIT gains might be higher among old people when obtained with the head hand position. In addition, the head velocity crucially determines the sensitivity of the vHIT. The false gain values might be caused by insufficient head stimulation velocity (<150°/s). However, stimulation with head velocities higher than 200°/s also causes inaccurate gain values. Therefore, the recommended optimal head stimulation velocity was 150–200°/s (3, 18). Besides, the relationships among the head velocity, gain, and age have been previously studied in healthy subjects. In the 140–160°/s and 180–200°/s head impulse velocity groups, the gain was stable up to the age of 80 years and up to the age of 70 years, respectively (19). In our study, we enrolled healthy subjects and patients who were less than 80 years old. And head stimulation velocity was 150–200°/s in all subjects. Therefore, the restricted condition of subjects may explain why no significant difference exists in the mean head velocity between the two head hand positions.

Patients with vestibular deficiency usually show reduced eye velocity and a retinal slip in response to head movements (gain reduction), which is one of the most effective error signals that drive adaptation on the VOR. Instead, a compensatory quick eye movement toward the target, called refixation saccades, will occur (3). Recently, several studies reported that refixation saccades with normal gain values can occur in patients with unilateral vestibulopathy (11, 20, 21). However, the hand position was not described in these studies. We cannot absolutely rule out the possibility of artifacts caused by different hand positions. Hence, in our study, we examined 67 UVN patients and had set the vHIT gain above 0.8 as the normal range in accordance with some previous reports (5, 9). When horizontal vHITs were performed with both hands on the top of the head in 67 UVN patients, 21 patients displayed refixation saccades with normal gain values. However, when the jaw hand position was combined in 67 UVN patients, 11 patients displayed refixation saccades with normal gain values and all the 11 patients were included in 21 patients. In our study, we had already verified that the head hand position can increase the VOR gain as compared with the jaw hand position. Therefore, this is one of the reasons for the presence of many cases of normal VOR with refixation saccades when using the head hand position. However, we still found some patients with normal VOR gains but with refixation saccades, irrespective of the hand positions used. After an acute vestibulopathy, the variation of the ipsilesional vHIT gain has been observed throughout the central compensation (22, 23). It is speculated that obtaining a normal VOR gain with refixation saccades would be possible when performing the head impulse test at some periods of the recovery (14).

### Limitations of the study

This study has several limitations. First, the number of subjects was insufficient. Second, we performed the vHIT using only on the GN-Otometrics vHIT system. At present, five video systems are commonly used (4). It remains unknown whether our results could be generalized to other similar recording systems. This means that our results need to be confirmed by using other systems. Third, we only found that the hand positions of the examiner affected the vHIT gains of healthy individuals and UVN patients. It is unknown whether the hand positions affect the vHIT in other vestibular disorders as well. Refixation saccades with gain values in the normal range are also reported following other vestibular disorders such as Ménière's disease, vestibular schwannoma, and cochlear implantation (20, 24). Further studies are necessary to investigate the effects of hand positions on VOR gain changes in different vestibular diseases.

## Conclusion

In this study, the jaw hand position increased the accuracy of the vHIT in determining the lesion side, resulting in avoided overhigh gain in the healthy side and decreased lesion side gain in most of the patients with UVN. Hence, we recommend that the jaw hand position should be routinely used in the clinical evaluation performed using the horizontal vHIT.

## Author contributions

WF designed the experiment, analyzed the data and wrote the article. FH, DW, and YB collected data. RZ prepared figures. XW and JH guided the study.

### Conflict of interest statement

The authors declare that the research was conducted in the absence of any commercial or financial relationships that could be construed as a potential conflict of interest.
